# MiR-143 inhibits endometrial cancer cell proliferation and metastasis by targeting *MAPK1*

**DOI:** 10.18632/oncotarget.21037

**Published:** 2017-09-16

**Authors:** Lei Chang, Dongya Zhang, Huirong Shi, Yangyang Bian, Ruixia Guo

**Affiliations:** ^1^ Department of Gynecology, The First Affiliated Hospital of Zhengzhou University, Zhengzhou, 450000 Henan, China; ^2^ Medical Research Center, The First Affiliated Hospital of Zhengzhou University, Zhengzhou, 450000 Henan, China

**Keywords:** endometrial cancer, miR-143, *MAPK1*, proliferation, migration

## Abstract

Endometrial cancer (EC) is one of the most commonly diagnosed gynecologic malignancies in the world, with the morbidity rate of over 7%. The mechanism of the pathogenesis has not been specifically elucidated to date, which is imperative for EC treatment. The aim of our study was to investigate the target relationship between miR-143 and mitogen-activated protein kinase 1 (*MAPK1*) and explore the effect of miR-143 on the endometrial cancers (EC) cells through targeting *MAPK1*. We collected EC tissues and adjacent tissues, and transfected miR-143 mimics and *MAPK1* siRNA into EC cells with lipofectamine. Reverse transcription-polymerase chain reaction (RT-PCR) and western blot were used to examine the expression of miR-143 and *MAPK1* mRNA and the protein expression of *MAPK1*. Cell counting kit-8, wound healing assay, flow cytometry and transwell assay were applied to examining the alteration of the proliferation, migration, cell cycle and invasion ability of EC cells. We predicted the targeting gene of miR-143 through bioinformatics analysis. MiR-143 was found under-expressed in EC tissues and cells. Overexpression of miR-143 or knockdown of *MAPK1* in human EC cell line HEC-1B inhibited the EC cell proliferation, migration and invasion and induced apoptosis. *MAPK1* was verified to be a target gene of miR-143. MiR-143 overexpression could effectively inhibit mRNA and protein expression of *MAPK1* in HEC-1B cells. Collectively, miR-143 might inhibit the proliferation, migration and invasion of EC cells, and promote the apoptosis of EC cells by suppressing *MAPK1*. These findings provided a view for new and potential therapeutic method for the clinical treatment of EC.

## INTRODUCTION

Endometrial cancer (EC) is one of the most commonly diagnosed gynecologic malignancies in the world [1]. EC was considered to have the highest incidence rate (approx. 7%) among the female genital malignant tumors and the incidence has been still on the rise [2]. EC was generally divided into types I (estrogen-dependent) and type II (estrogen-independent) [3]. The vast majority of endometrial carcinomas (80%) belong to type I, only second to atypical endometrial hyperplasia [4]. he EC type I is associated with obesity, infertility, insulin resistance and estrogen therapy, while the EC Type II includes endometrial serous carcinoma, clear cell carcinoma, squamous cell carcinoma, mucinous adenocarcinoma and other types of cancers. High malignancy, poor prognosis, early-stage metastasis and worse clinical outcome often embody in the EC Type II [5]. Previous research has already revealed that several aberrant gene expressions and transduction pathways forebode the initiation of EC [6]. As EC is a multi-factorial disease, the deep mechanism of the pathogenesis has not been specifically elucidated. Therefore, it is of paramount importance to understand the mechanism of EC for the disease prevention and clinical diagnosis and treatment.

MicroRNAs (miRNAs) are a group of small, single-stranded, endogenous and non-coding RNAs [7, 8], which can help regulate different aspects in cancers pathological process, including invasion, migration, proliferation, differentiation, apoptosis and senescence [9–11]. MiRNAs regulate the expression of the target gene by targeting 3’UTR of messenger RNA, ultimately leading to either mRNAs repression or transcription degradation at the posttranscriptional and transcriptional level [12]. In the last few years, substantiated evidence has showed that miRNAs were involved in many cellular processes through the regulation of specific target genes [13]. On the one hand, miRNAs could serve as oncogenes and promoted the process of tumorigenesis [14, 15]. For example, miR-9 could promote tumor metastasis by down-regulating the expression of E-cadherin in esophageal squamous cell carcinoma [16]. On the other, miRNAs also function as tumor suppressors and inhibit the progression of cancers. For example, miR-27a could directly target *KRAS* to inhibit cells proliferation in esophageal squamous cell carcinoma [17]. MiR-99a could suppress the metastasis of human lung tumor cells by targeting the AKT1 signaling pathway [18]. However, the function and the molecular mechanism of miR-143 in the regulation of EC malignant behavior remain to be further investigated.

Mitogen-activated protein kinase 1 (*MAPK1*) belongs to the MAP kinase family. MAP kinase, also known as extracellular signal-regulated kinases (ERKs), could act as a binding point for numerous biochemical signals [19, 20]. Besides, ERKs are also involved in a wide range of cellular processes, such as differentiation, proliferation, transcription development and regulation [21]. Moreover, the activation of ERKs requires upstream kinases phosphorylation. Once activated, this kinase could translocate to the nucleus of the activated tumor cells, that is, phosphorylates nuclear targets [22, 23]. Previous study has demonstrated that this protein could function as a transcriptional repressor independently because of its kinase activity [24]. Some studies also revealed that *MAPK1* played an important role in tumors progression and different tumors cellular processes [25–27]. In addition, several researchers found that *MAPK1* could serve as potential target gene for miRNAs and miR-378 inhibited prostate cancer cell growth through targeting *MAPK1* [28]. MiR-143 has been reported to participate in carcinogenesis by influencing MAPK pathway such as pancreatic ductal adenocarcinoma, bladder cancer and acute myeloid leukemia [29–31]. In addition, Ning *et al.* showed that MAPK1 can be directly targeted by miR-143 during human keratinocyte differentiation [32]. MAPK1 has also been reported to be a common target of miR-143 in renal cell carcinoma [33]. However, few researches were conducted to analyze the relationship between *MAPK1* and miR-143 in EC. In our study, we demonstrated that miR-143 could affect EC cells proliferation, migration and invasion through targeting *MAPK1*.

## RESULTS

### MiR-143 expression was downregulated in EC tissues and cells

**Figure 1:**
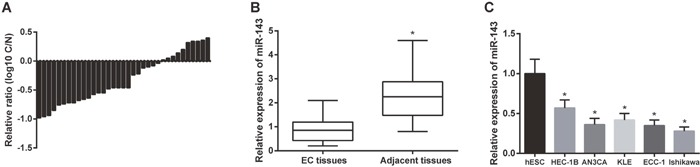
The expression levels of miR-143 were down-regulated in EC tissues and cell lines **(A)** The expression levels of miR-143 in tissue samples were down-regulated compared with normal tissues. C: EC tissues, N: normal tissues. **(B)** The expression levels of miR-143 in EC tissues were much lower than that in normal tissues. ^*^ *P*<0.05 compared with EC tissues. **(C)** The expression levels of miR-143 in EC cell lines (HEC-1B, AN3CA, KLE, ECC-1, and Ishikawa) was observably lower than that in normal cell line (hESC). ^*^ *P*<0.05 compared with hESC group.

To study the expression of miR-143 in EC, we measured the expression of miR-143 in 35 EC tissues and the adjacent tissues using RT-PCR. The results indicated that miR-143 expression was significantly downregulated in the EC tissues in comparison with adjacent tissues (*P* < 0.05, Figure 1A and 1B). Besides, the miR-143 expression in EC cell lines (HEC-1B, AN3CA, KLE, ECC-1and Ishikawa) was significantly lower than normal cells (hESC, *P* < 0.05, Figure 1C). Given that the expression of miR-143 in HEC-1B cells was relatively higher in comparison with the other four EC cell lines, we selected HEC-1B cell line for our further study.

### Overexpression of miR-143 reduced cell viability and cell cycle, inhibited migration and invasion ability of EC cells and promoted cell apoptosis

**Figure 2:**
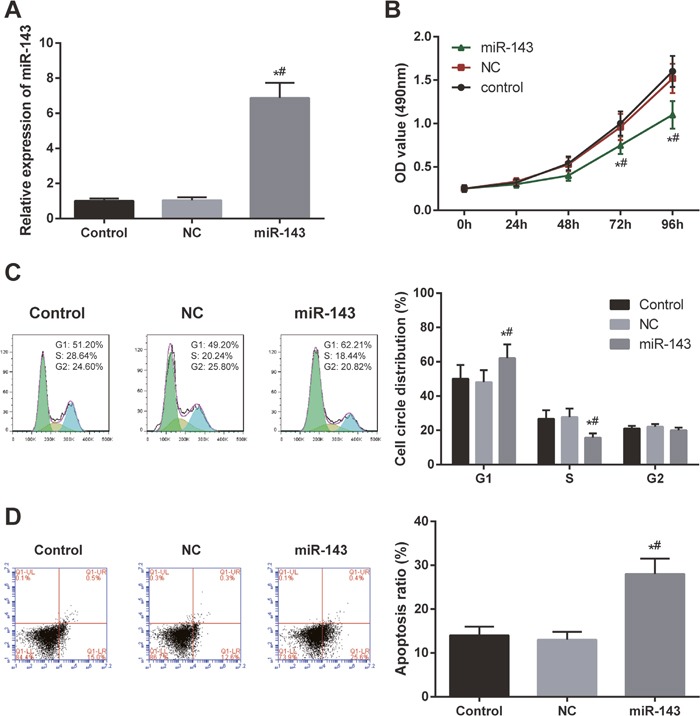
Overexpression of miR-143 inhibited cell proliferation and induced apoptosis of the cells **(A)** The expression of miR-143 in HEC-1B cells dramatically increased after transfection with miR-143 mimics. **(B)** Overexpression of miR-143 suppressed HEC-1B cell viability. **(C)** Overexpression of miR-143 induced EC cell cycle arrest. X axis indicates DNA content and Y axis indicates cell number. Color green: G1 phase; yellow: M phase; blue: G2/M phase. **(D)** Overexpression of miR-43 promoted EC cell apoptosis observed by FCM. X axis indicates AnnexinV fluorescence intensity and Y axis indicates PI fluorescence intensity. ^*^ *P*<0.05 relative to control group, ^#^ *P*<0.05 relative to NC group.

The expression of miR-143 was detected by RT-PCR. After HEC-1B cells were transfected with miR-143 mimics, miR-143 expressions were upregulated, and miR-143 expression in miR-143 mimics group was higher than that in blank control group and negative control (NC) group (*P* < 0.05, Figure 2A). Compared with control group and NC group, cell viabilities in miR-143 group were significantly suppressed (*P* < 0.05, Figure 2B). Cell cycle and apoptosis of EC cells was assessed using FCM. The results have shown that in miR-143 group, cell cycle arrested at G1 phase and a constant accumulation of apoptotic cell was detected (both *P* < 0.05, Figure 2C and 2D).

**Figure 3:**
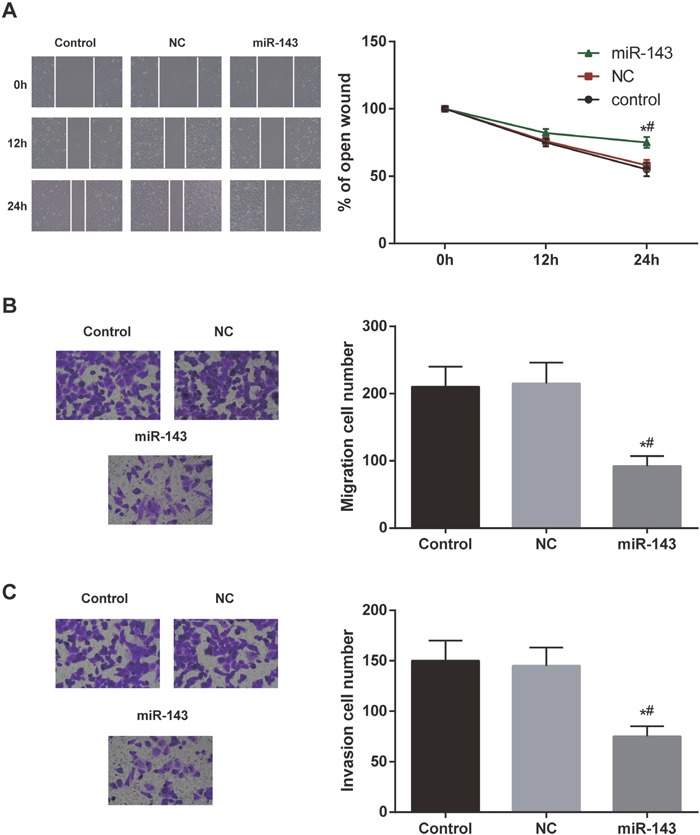
Overexpression of miR-143 inhibited cell invasion and migration **(A)** The open wound area of EC cells in miR-143 group was much larger than that in control group and NC group. **(B)** The number of migration cellS in miR-143 group was relatively fewer compared with control group and NC group. **(C)** The number of invasion cells in miR-143 group was more than that in control group and NC group detected. ^*^ *P*<0.05 compared with control group, ^#^ *P*<0.05 compared with NC group.

The migration and invasion abilities of the EC cells were examined in each group by wound healing assay and transwell assay. As shown in the Figure 3, miR-143 mimics group displayed weaker migration and invasion abilities of EC cells in comparison with control group and NC group (both *P*<0.05).

### Protein and mRNA expression of *MAPK1* were observably suppressed by overexpression of miR-143

**Figure 4:**
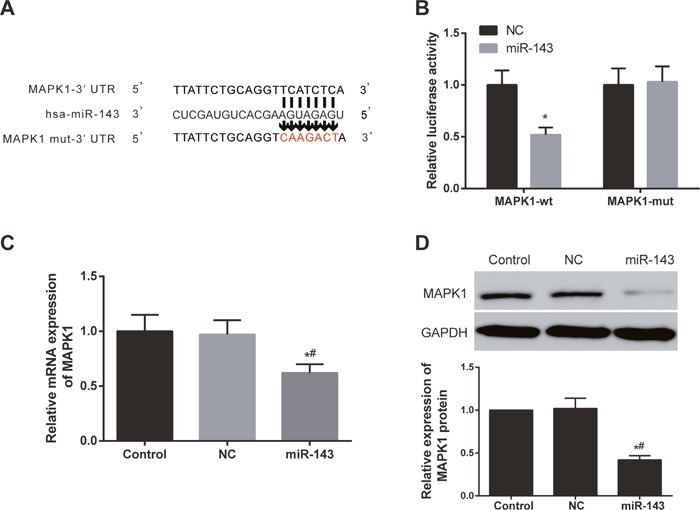
MiR-143 directly targeted and regulated MAPK1 in EC cells **(A)** MiR-143 could directly target 3’UTR of *MAPK1*. **(B)** The luciferase activity of luc-*MAPK1*-wt was suppressed by miR-143 overexpression, but there was significant difference in luc-*MAPK1*-mut. ^*^ *P*<0.05 compared with NC group. **(C)** Overexpression of miR-143 suppressed *MAPK1* expression in EC cells. **(D)** Overexpression of miR-143 inhibited the expression of *MAPK1*. ^*^ *P*<0.05 compared with control group, ^#^ *P*<0.05 compared with NC group.

To validate that *MAPK1* was a direct target of miR-143, two types of MAPK1 vectors including wild type (WT) luc-*MAPK1* and mutation type luc-*MAPK1*-mut were constructed (Figure 4A). Overexpression of miR-143 could inhibit the luciferase activity of luc-*MAPK1*, but had no effect on luc-*MAPK1*-mut (Figure 4B). This also indicated that miR-143 could directly target 3’UTR of *MAPK1* and inhibited the transcriptional activity of *MAPK1*. The results of RT-PCR and western blot both revealed that protein and mRNA expressions of *MAPK1* were suppressed by overexpression of miR-143 (Figure 4C and 4D).

### Down-regulation of *MAPK1* expression could inhibit the proliferation ability of EC cells and induce apoptosis as well as change the cell cycle distribution

**Figure 5:**
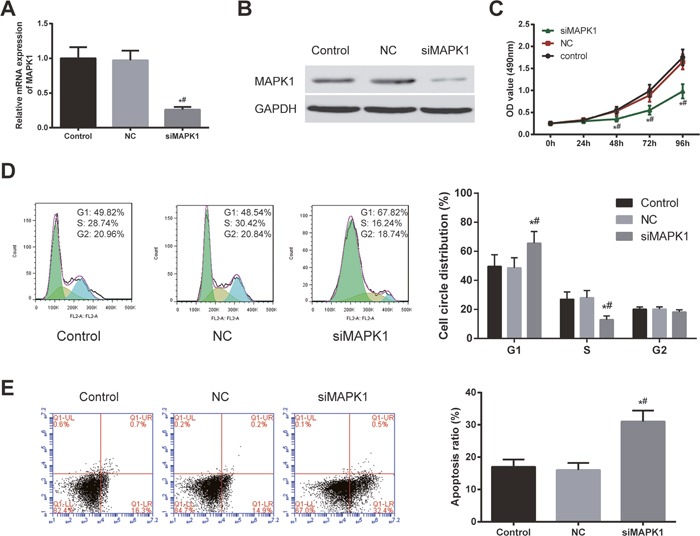
Down-regulation of MAPK1 expression inhibited EC cell proliferation and induced cell cycle arrest and cell apoptosis **(A)** The expression of *MAPK1* in HEC-1B cells after transfection with si-*MAPK1* significantly decreased. **(B)** The expression of *MAPK1* was down-regulated after transfection with si-*MAPK1*. **(C)** The EC cell viability in siMAPK1 group was much lower than that in control group and NC group confirmed. **(D)** The number of EC cells arrested in G1 stage increased after transfected with si-*MAPK1* for 48 h. X axis indicates DNA content and Y axis indicates cell number. Color green: G1 phase; yellow: M phase; blue: G2/M phase. **(E)** Theapoptosis of EC cells significantly enhanced after transfected with *si-MAPK1* for 48 h. X axis indicates AnnexinV fluorescence intensity and Y axis indicates PI fluorescence intensity. ^*^ *P*<0.05 compared with control group, ^#^ *P*<0.05 compared with NC group.

The expression of *MAPK1* in HEC-1B cells was knocked down by siRNA. The *MAPK1* expression was evaluated using RT-PCR and western blot. The mRNA and protein expressions were down-regulated in HEC-1B cells after transfected with *MAPK1*-siRNA (*P*<0.05, Figure 5A and 5B). According to CCK-8 assay results, the proliferation rate of HEC-1B cells in si*MAPK1* group was much lower than that in the other two groups at 48 h, 72 h and 96 h (*P*<0.05, Figure 5C). The cell cycle distribution of HEC-1B cells were changed after inhibiting *MAPK1*, the number of cells in G1 phase increased and cells in S phase reduced (Figure 5D). In addition, the inhibition of *MAPK1* could induce the apoptosis of HEC-1B cells (*P*<0.05, Figure 5E).

### The inhibition of *MAPK1* expression in EC cells could suppress the migration and invasion abilities of the cells

**Figure 6:**
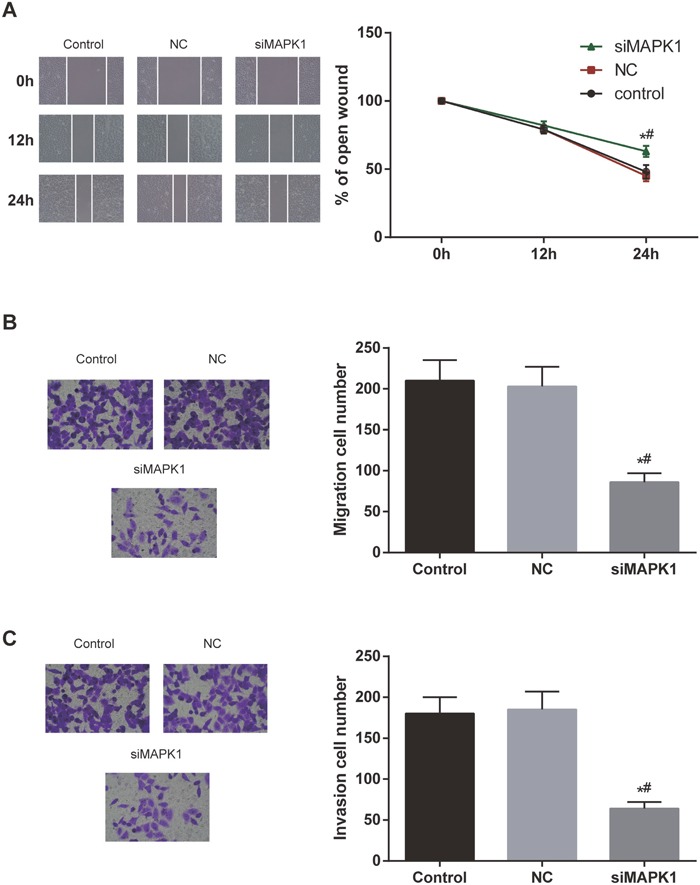
The inhibition of MAPK1 expression in EC cells suppressed the migration and invasion abilities of the cells **(A)** The open wound area in si-*MAPK1* group was larger than that in control group and NC group. **(B, C)** The migration and invasion ability of EC cells significantly decreased after transfection with si-*MAPK1*. ^*^ *P*<0.05 compared with control group, ^#^ *P*<0.05 compared with NC group.

In wound healing assay, the wound healing ability of HEC-1B cells was found to be significantly inhibited 24 h after transfection with *MAPK1* (both *P*<0.05, Figure 6A). Furthermore, transwell assay also displayed that inhibition of *MAPK1* expression in HEC-1B cells could suppress the migration and invasion abilities of the cells (both *P*<0.05, Figure 6B and 6C).

## DISCUSSION

In the current study, miR-143 was found under-expressed in EC tissues and cells. Overexpression of miR-143 or knockdown of *MAPK1* in human EC cell line HEC-1B inhibited the EC cell proliferation, migration and invasion and induced apoptosis. *MAPK1* was verified to be a target gene of miR-143. MiR-143 overexpression could effectively inhibit mRNA and protein expression of *MAPK1* in HEC-1B cells.

EC is one of the most widespread gynecologic tumors among women around the world [34]. Many types of aberrant molecular expressions, including oncogenes and tumor suppressors, are related to progression and tumorigenesis of EC [35]. For a molecular mechanism, multiple regulators of gene expression have been founded and miRNA plays a vital role in the regulation of gene expressions. Recent findings have found that miR-143 family members exerted influence on multiple features of tumor cells through different pathways and targets. For instance, Liu *et al*. found that miR-143 could mediate the apoptotic process in osteosarcoma cells by manipulating the expression of Bcl-2. Mao *et al*. revealed that miR-143, as a tumor suppressor, could inhibit the expression of family with sequence similarity 83 (FAM83F) in esophageal squamous cell carcinoma (ESCC). Besides, Liu *et al*. found that miR-143 could inhibit cell invasion and proliferation through down-regulating TLR2 expression in hepatoma cells [36–38]. These studies indicate that miR-143 could possibly suppress EC progression or development by silencing some gene. In our study, the down-regulation of miR-143 in EC tissues and cells indicated that miR-143 could function as a tumor suppressor in EC. We have confirmed that miR-143 could efficiently down-regulate *MAPK1* expression in EC cells. Thus, we speculate that miR-143 could probably hinder EC carcinogenesis by inhibiting *MAPK1*.

*MAPK1* can be targeted by various miRNAs in various cancers. For instance, Kouhkan *et al*. found that cell cycle arrest of glioblastoma multiforme (GBM) cancer cells was induced by miR-192-1's targeting *MAPK1*. Chen *et al*. revealed that miR-378 suppressed prostate cancer cell growth through down-regulation of *MAPK1 in vitro* and *in vivo*. Yan *et al*. demonstrated that deregulated miR-335 that targets *MAPK1* was implicated in poor outcome of pediatric acute lymphoblastic leukemia [28, 39, 40]. *MAPK1* is the core protein of the MAPK/ERK pathway. As we know, *MAPK1* could manipulate cell biological function by modulating downstream substrates of the MAPK/ERK signaling pathway, such as Cyclin D1 and c-MYC [41]. Up to now, nearly one-third of human cancers revealed that MAPK/ERK pathway was abnormally activated and played an essential role in their pathogenesis [42]. Since the MAPK/ERK signaling pathway plays a vital role in the development of tumor, numerous researches have focused on the potential therapeutic implication. For instance, Sorafenib, a tyrosine kinase inhibitor (TKI), could inhibit papillary thyroid carcinoma growth by down-regulating MAPK/ERK signaling pathway [43]. Moreover, miRNAs have been confirmed to regulate target genes of MAPK/ERK pathway, to some extent, demonstrating their important roles in manipulation of the signaling cascade. Liu *et al*. revealed that miR-524-5p could inhibit cell migration and tumor proliferation and suppressed their activities in melanoma by targeting *MAPK1* and BRAF through MAPK/ERK signaling pathway [44]. Another research showed that miR-133a inhibited the MAPK/ERK cascade by targeting the upstream protein LASP1, which could suppress tumor metastasis and growth in colorectal cancer [45]. All in all, we could conclude that miR-143 could suppress EC cell migration, invasion and proliferation by targeting down *MAPK1*, which therefore suppresses MAPK/ERK signaling.

In the current study, we first demonstrated the molecular mechanism of miR-143 in influencing EC cells through targeting *MAPK1*. MiR-143 was found to be capable of suppressing proliferation, migration and invasion and cell cycle process of HEC-1B cells, and inducing apoptosis via downregulation of *MAPK1*, which not only proved our previous conjuncture was right, but provided some valuable evidence for the intensive study on the subject. However, it was not sufficient to completely reach the conclusion through *in vitro* experiments only, which was also a limitation of our study. Therefore, our later study would further substantiate the conclusion by conducting *in vivo* experiments.

In conclusion, our study showed that overexpression of miR-143 could suppress proliferation, migration and invasion and cell cycle process of HEC-1B cells, and induce apoptosis by targeting *MAPK1*. These results revealed that miR-143 functions as a tumor suppressor and might play an important role in the inhibition of EC cell proliferation and metastasis. These findings not only laid foundation for the in-depth study on the molecular mechanism of mRNA, but may provide a new and potential therapeutic method for the clinical treatment of EC.

## MATERIALS AND METHODS

### Samples

We collected tissue samples from 35 EC patients in the First Affiliated Hospital of Zhengzhou University. Before surgery, radiotherapy or chemotherapy was not applied to any one of the patients. The adjacent non-neoplastic tissue was over 5 cm distance from the tumor's margin. All tissue specimens were frozen immediately using liquid nitrogen, after which tissues were conserved under 80 degree centigrade. The collection of EC tissue samples in this study was approved by the First Affiliated Hospital of Zhengzhou University Ethics Committee. Meanwhile, human endometrial stromal cell line (hESC) and endometrial carcinoma cell lines (HEC-1B AN3CA, KLE, ECC-1, and Ishikawa) cells in the study were bought from the Cell Center of Chinese Academy of Medical Sciences. The cells were cultured in dulbecco's modified eagle medium (DMEM; Sigma, St. Louis, MO, USA) culture medium with 10% fetal bovine serum (FBS; Invitrogen, Gaithersburg, MD, USA), 95% humidity and 5% CO_2_ at 37 degrees centigrade.

### Cell transfection

The vectors containing negative control, miR-143 mimics and *MAPK1* siRNA were bought from Dharmacon Corporation in USA. Human EC cells were incubated in culture plate with 50%-70% density of DMEM without fetal calf serum (FCS; Invitrogen, Gaithersburg, MD, USA) and cultured one day before transfection. Vectors were transferred into cells through lipofectamine Dharma FECT I in accordance with the manufacturer's protocols.

### Cell counting kit-8 (CCK8)

Cells were collected and dissolved with 0.25% pancreatic enzymes after transfection for 16 h. Then the cells were resuspended in complete medium and plated at a density of 0.8×10^5^ cells/well on 96-well plates, and repeated five times in every group. CCK8 (Beyotime, Jiangsu, China) was added into the plates along the wall of the wells, shaken for blending and cultured at 37 degree centigrade for 2 h. Wavelength absorbance of optical density (OD) value was set at 490 nm for each well on automatic enzyme-linked immune detector. Cell proliferation activity was determined with CCK8 method after transfection for 0 h, 24 h, 48 h, 72 h and 96 h.

### Flow cytometry (FCM) assay

After transfected for 16 h, HEC-1B cells were collected. The cells were washed twice with phosphate buffered saline (PBS) and then were resuspended with PBS. Later, 100 μg/ml RNase A was dropped into cells, which were incubated at 37 degree centigrade for 30 min. Finally, a total of 50 μg/ml phosphate (PI) was added and cell cycle was analyzed through FCM (BD Biosciences, New York, USA).

After being transfected for 48 h, the cells were digested by trypsin, and then the cells were fixed and washed twice with PBS. The EC apoptosis was evaluated using a PE Annexin V apoptosis detection kit I. 5×10^5^ EC cells were incubated with binding buffer and respectively added annexin V-PE/7-ADD. After being cultured 15 min at room temperature, 200 μL binding buffer was added and cell apoptosis was evaluated by means of FCM (BD Biosciences, New York, USA).

### Wound healing assay

Wound healing assay was performed to examine and evaluate the migration ability of the cells. Cells were seeded in twelve-well plates at the density of 2×10^5^ cells per well. At 80-90% confluence, the monolayer of cells was scratched with a sterile 200 ml micropipette tip. The scratched cells were washed with serum-free medium three times. After being removed from the cellular debris, the cells were incubated for 0 h, 12 h, 24 h, and closed area of the wound was observed under an optical microscope (Nikon, Japan).

### Transwell assay

Matrigel was melted at 4 °C overnight, and diluted with precooling serum-free medium to 1 mg/mL. Then 40 μL Matrigel was added to the upper one of chambers and incubated at 37 degree centigrade for 4-5 h. In the upper chamber, we added a total of 2×10^5^ EC cells in 200 μL serum-free medium. In the lower chamber, we added 600 μL of DMEM medium containing 10% FBS. After incubated at 37 degree centigrade and 5% CO_2_ for 24 h, cotton swabs were used to slightly wipe off the cells which failed to pass through and still remained on the membrane surface. The cells which invaded or migrated to the lower chamber were fixed with methanol for 10 min and stained with 0.1% crystal violet for 5 min. Finally, the number of invasion cells was observed and counted on the lower surface under a microscope.

### Dual luciferase reporter assay

*MAPK1* 3’UTR with miR-143 binding site was amplified to psiCHECK-2 luciferase vector (Promega, Madison, WI, USA) as luc-*MAPK1* vector. T Luc-*MAPK1*-mut vector with *MAPK1* 3’UTR mutation without miR-143 binding site was constructed by XL Site-directed Mutagenesis Kit (Qiagen, Hilden, Germany). Cells were co-transfected with Luc-*MAPK1*-wt/Luc-*MAPK1*-mut and miR-143/negative. After transfected for 48 h, cell gene activity was tested by dual luciferase assay kit (Promega, Wisconsin, USA). Turner Designs Spreadsheet Interface Version 2.0.1 was used for calculation and analysis.

### Real-time PCR (RT-PCR)

Total RNA from the EC cells or tissues was extracted using Trizol Reagent (Invitrogen, Carlsbad, CA) following the instructions of manufacturer. Total RNA was reversely transcribed into cDNA using TIANScript RT Kit (Tiangen biotech, Beijing, China). Real-time PCR was carried out on a THUNDERBIRD SYBR® qPCR Mix (Toyobo, Japan). The RT-PCR was performed with CFX96 Touch RT-PCR Detection System (Bio-Rad). *U6* and *GAPDH* were employed as reference genes to normalize the expression of miRNA and mRNA, respectively. The primers used for RT-PCR were listed in Table 1.

**Table 1:** The sequences of primers used in RT-PCR

cDNA		Primer sequences
miR-143	F	5’-AGTGCGTGTCGTGGAGTC-3’
	R	5’-GCCTGAGATGAAGCACTGT-3’
U6	F	5’-CTCGCTTCGGCAGCACA-3’
	R	5’-AACGCTTCACGAATTTGCGT-3’
MAPK1	F	5’-AGGCTGTTCCCAAATGCT-3’
	R	5’-CGTCACTCGGGTCGTAAT-3’
GAPDH	F	5’-GAAATCCCATCACCACTTCCAGG-3’
	R	5’-GAGCCCCAGCCTTCTCCATG-3’

F: forward primer; R: reverse primer.

### Western blot

Total proteins were extracted from the cells by RIPA buffer (Sigma-Aldrich, St. Louis, MO). BCA kit (Beyotime Biotechnology, Haimen, China) was employed to examine the protein concentrations. Then the proteins were separated from sodium dodecyl sulfate polyacrylamide gel electrophoresis (SDS-PAGE; Bio-Rad, Hercules, CA, USA) and transferred into polyvinylidene fluoride PVDF (Invitrogen, Gaithersburg, MD, USA) membranes. The membranes were washed off with TBST for 2 min and blocked in 5% non-fat milk for 2 h at room temperature. After blocked, the membranes were incubated with mouse anti-MPK1 and mouse anti-GAPDH primary antibodies (Abcam, Southampton, UK) followed by incubation with HRP-conjugated secondary antibody (Abcam, Southampton, UK) for 1 h at room temperate. The relative band density was determined using an enhanced chemiluminescence (ECL) detection system (Amersham Corp., Amersham, U.K.). Lab Work4.5 was used to examine the OD of the protein bands.

### Statistical analysis

SPSS 21.0 software (IBM Corporation, New York, NY, USA) was used for performing statistical analyses. All data are presented as the mean ± SD. Differences were analyzed by t-test or one-way analysis of variance. *P*<0.05 was considered statistically significant.

## Abbreviations

CCK8: Cell counting kit-8
DMEM: dulbecco's modified eagle medium
EC: endometrial cancer
FCS: fetal calf serum
FCM: flow cytometry
GBM: glioblastoma multiforme
miRNAs: microRNAs
NC: negative control
PBS: phosphate buffered saline
PI: phosphate
WT: wild type
